# Barriers, supports, and effective interventions for uptake of human papillomavirus- and other vaccines within global and Canadian Indigenous peoples: a systematic review protocol

**DOI:** 10.1186/s13643-018-0692-y

**Published:** 2018-03-02

**Authors:** Kelly J. Mrklas, Shannon MacDonald, Melissa A. Shea-Budgell, Nancy Bedingfield, Heather Ganshorn, Sarah Glaze, Lea Bill, Bonnie Healy, Chyloe Healy, Juliet Guichon, Amy Colquhoun, Christopher Bell, Ruth Richardson, Rita Henderson, James Kellner, Cheryl Barnabe, Robert A. Bednarczyk, Angeline Letendre, Gregg S. Nelson

**Affiliations:** 1 Research Innovation and Analytics, Alberta Health Services, Edmonton, Canada; 2 Department of Community Health Sciences, Cumming School of Medicine, University of Calgary, Calgary, Canada; 3 Faculty of Nursing, University of Alberta, Edmonton, Canada; 4 Arnie Charbonneau Cancer Institute, University of Calgary, Calgary, Canada; 5 Department of Oncology, Cumming School of Medicine, University of Calgary, Calgary, Canada; 6 Health Sciences Library, Libraries and Cultural Resources, University of Calgary, Calgary, Canada; 7 Department of Obstetrics and Gynaecology, Cumming School of Medicine, University of Calgary, Calgary, Canada; 8 Alberta First Nations Information Governance Centre, Calgary, Canada; 9 Analytics and Performance Reporting Branch, Alberta Health, Edmonton, Canada; 10 Health Canada First Nations and Inuit Health Branch, Edmonton, Canada; 11 Department of Paediatrics, Cumming School of Medicine, University of Calgary, Calgary, Canada; 12 Hubert Department of Global Health, Rollins School of Public Health, Emory University, Atlanta, GA USA; 13 Alberta Cancer Prevention Legacy Fund, Edmonton, Canada

**Keywords:** Barriers, Supports, Human papillomavirus (HPV), Vaccination, Indigenous populations, Acceptance, Hesitancy, Uptake, Cancer prevention

## Abstract

**Background:**

Despite the existence of human papilloma virus (HPV) vaccines with demonstrated safety and effectiveness and funded HPV vaccination programs, coverage rates are persistently lower and cervical cancer burden higher among Canadian Indigenous peoples. Barriers and supports to HPV vaccination in Indigenous peoples have not been systematically documented, nor have interventions to increase uptake in this population. This protocol aims to appraise the literature in Canadian and global Indigenous peoples, relating to documented barriers and supports to vaccination and interventions to increase acceptability/uptake or reduce hesitancy of vaccination. Although HPV vaccination is the primary focus, we anticipate only a small number of relevant studies to emerge from the search and will, therefore, employ a broad search strategy to capture literature related to both HPV vaccination and vaccination in general in global Indigenous peoples.

**Methods:**

Eligible studies will include global Indigenous peoples and discuss barriers or supports and/or interventions to improve uptake or to reduce hesitancy, for the HPV vaccine and/or other vaccines. Primary outcomes are documented barriers or supports or interventions. All study designs meeting inclusion criteria will be considered, without restricting by language, location, or data type. We will use an a priori search strategy, comprised of key words and controlled vocabulary terms, developed in consultation with an academic librarian, and reviewed by a second academic librarian using the PRESS checklist. We will search several electronic databases from date of inception, without restrictions. A pre-defined group of global Indigenous websites will be reviewed for relevant gray literature. Bibliographic searches will be conducted for all included studies to identify relevant reviews. Data analysis will include an inductive, qualitative, thematic synthesis and a quantitative analysis of measured barriers and supports, as well as a descriptive synthesis and quantitative summary of measures for interventions.

**Discussion:**

To our knowledge, this study will contribute the first systematic review of documented barriers, supports, and interventions for vaccination in general and for HPV vaccination. The results of this study are expected to inform future research, policies, programs, and community-driven initiatives to enhance acceptability and uptake of HPV vaccination among Indigenous peoples.

**Systematic review registration:**

PROSPERO Registration Number: CRD42017048844

**Electronic supplementary material:**

The online version of this article (10.1186/s13643-018-0692-y) contains supplementary material, which is available to authorized users.

## Background

### Rationale

Persistent infection with human papillomavirus (HPV) strains 16 and 18 can cause cervical and other cancers [1]. The prevalence of these strains can vary by socioeconomic factors, including geography and ethnicity [2, 3]. There are well-documented inequities in cancer prevention, disease burden, and subsequent treatment, for First Nation (FN), Inuit, and Métis peoples in Canada [2, 4–10]. While acknowledging that there is heterogeneity across and within each of these peoples, we respectfully refer to them ‘Indigenous unless otherwise specified. The prevalence of HPV infection and cervical cancer is significantly higher [11–23] and HPV vaccination rates lower among Indigenous peoples [23]. This inequity exists despite the availability of vaccines with demonstrated safety profiles and efficacy, and funded vaccination programs [24–26].

Enhancing HPV vaccination is a potentially high-impact, low-cost opportunity for cancer risk reduction among Indigenous peoples. As part of a tri-phase study in First Nations people to (1) establish baseline HPV vaccination rates, (2) systematically identify and validate the barriers and supports to HPV vaccination, and (3) comprehensively map documented and reported supports and barriers to HPV vaccination to develop a theory-informed, context-appropriate knowledge translation intervention, this synthesis aims to identify documented barriers, supports, and intervention strategies for HPV vaccination in Canadian and global Indigenous populations.

### Vaccine acceptability, hesitancy, and uptake

Despite its success as a public health intervention [27–34], vaccination in many countries still falls short of levels needed to ensure community protection [30, 35–37]. Even where vaccination rates are high, ongoing acceptance remains a vulnerability [35], as evidenced by significant variability among different groups within high vaccine-coverage countries [30, 37]. Measuring vaccination rates can be challenging, requiring different metrics to quantify impact. “Vaccine coverage” (e.g., the proportion of the target population who have received the vaccine) is the typical population-level measure of protection [30, 37–39], while “vaccine acceptability” and “uptake” are more common, individual-level measures. “Vaccine acceptability” reflects individuals’ willingness to accept vaccination, typically assessed as attitudes, beliefs, and/or intentions [40]. A more commonly used term, “vaccine hesitancy”, refers to a delay in acceptance or refusal of vaccines despite the availability of vaccine services [41]. “Vaccine uptake” reflects the behavior of getting vaccinated [37]. Understanding the barriers and supports to vaccine acceptability/hesitancy, moving from acceptance to uptake, and uptake from multiple perspectives (e.g., recipient, parents/guardians, health care providers, policy makers) [25], is a critical first step to tailoring strategies to enhance vaccination [37, 42].

### Barriers and supports to vaccine acceptability and uptake

General barriers and supports to vaccine acceptability and uptake of childhood vaccines have been documented [41, 43, 44]. The most common factors include socio-demographic characteristics (e.g., race, marital status, maternal age/education, family size/income) [45–50], knowledge and beliefs (e.g. fear of side effects, lack of perceived susceptibility to disease) [45, 46, 49, 50], trust in the establishment (e.g., trust in the medical community or government) [46, 49–51], social and cultural norms (e.g., family, religious, or community influences) [45, 49], worldview regarding health (e.g., preference for natural interventions) [45, 49], healthcare provider influence (e.g., provider recommendations and/or knowledge) [49, 50], competing priorities (e.g., social support, child care) [50, 52, 53], concern about vaccination processes (e.g., vaccination pain or needles) [46, 49–51], and access to services (e.g., clinic hours and location) [48–50]. Some of these factors are more prominent in specific sub-populations and/or for specific vaccines [30, 40].

### HPV vaccine-specific barriers, supports, and effective interventions

In Canada, the HPV vaccine was introduced into national and regional vaccination programs targeting pre-adolescents in school settings [54], to prevent cancers caused by HPV [55, 56]. Vaccines preventing HPV infection demonstrate long-term effectiveness and acceptable safety profiles [24]. However, a number of issues specific to the HPV vaccine influence its acceptability and uptake in the general population; these factors are well described in systematic reviews [25, 26, 57–61] and include knowledge and perceived risk of HPV disease, misinformation about vaccine safety, cost, misperceptions that the vaccine induces sexual promiscuity, and multi-dose vaccine series delivery. Additional challenges in the school setting include the information and consent process, vaccination setting, and student anxiety regarding privacy and pain during vaccination [62]. In high-income countries, access to and receipt of the HPV vaccine is determined by factors that influence policy makers, health care professionals, and parental decision-making, including financial constraints, social norms and values concerning sexual activity, and trust in vaccination programs and health care providers [61]. Further, barriers, and supports may be context-specific; for example, access to HPV vaccine may be facilitated by universal coverage health care systems or hindered by parents’ cultural or religious perceptions about sexual activity [61]. Much of the literature reports barriers and supports to HPV vaccine acceptability, as reported by parents, health care providers, and policy makers [25], rather than vaccine recipients. This gap in self-reported data hinders effective, evidence- and theory-informed intervention mapping [63].

A recent systematic review [25] summarized the nature and frequency of adolescent females’ self-reported barriers to HPV vaccination. These factors included cost, perceived lack of need, concerns about safety and side effects, perceived efficacy, perceived relative benefit, vaccination logistics, fear of needles, vaccine novelty, perception that sexual inactivity precludes the need for vaccination, negative physician-related recommendations, social norms, inability to discuss the vaccine with parents, lack of awareness or information, anti-vaccination views, subjective norms, pregnancy (or trying to conceive), self-efficacy, mistrust of pharmaceutical companies, and the perception that alternative strategies are available. Cost and the perception that HPV vaccination leads to increased sexual activity were previously identified as vaccination barriers [60]. Adolescent females’ self-reported supports to HPV vaccination included perceived benefit, positive health care provider recommendations, social norms, parental endorsement, perceived risk, cost-free access, subjective norms, perceived severity, positive attitude toward vaccine or reported personal importance of vaccine, self-efficacy, and vaccine efficacy [25, 60].

Although a recent systematic review reported a lack of consistency and detailed reporting of interventions to increase HPV vaccine uptake [64], it found that the nature and magnitude of effect for national and international strategies (e.g., single and multifaceted behavioral [targeted at patient, provider and patient-provider] and environmental [local and national level policies]) were documented. It also revealed that environmental interventions (e.g., school-based vaccine administration) achieved the highest vaccination rates, but noted a lack of data for differentiating subpopulations. The need was underscored for specific strategies and interventions to target underserved populations, including Indigenous youth [64, 65].

### Barriers and supports to HPV vaccine acceptability and uptake in indigenous peoples

HPV vaccine coverage levels in the general population of pre-adolescents may hide sub-populations with lower coverage, including Indigenous peoples who are known to have higher HPV infection rates [2, 11, 66], lower cervical cancer screening rates [13, 14, 67], higher rates of invasive cervical cancer [15, 68], higher rates of cervical cancer-related hospitalizations [16], and lower rates of cervical cancer survival [17, 18, 69]. There is a paucity of data on HPV vaccination coverage in Indigenous peoples; however, it is well known that other childhood vaccinations in these groups are typically below that of the general population [52, 70–76]. Ongoing work in Canada [77] and Australia [78] strives to accurately track coverage levels, but an important companion to this work is a more detailed understanding of the barriers and supports to vaccination in Indigenous peoples. Moreover, generic interventions aimed at improving vaccine uptake in the general population of pre-adolescent children may require modifications or redesign for Indigenous peoples [79, 80], particularly given the multijurisdictional complexities in health services delivery for those living on reserve. In addition, context and pragmatics (e.g., differing HPV vaccination programs in municipal schools versus on-reserve schools) may impact coverage levels [81].

The evidence compiled thus far on the barriers, supports, and interventions for HPV vaccination reveals considerable gaps in data for Indigenous peoples. Even so, any evidence on vaccination in this population may provide important guidance for future work. To our knowledge, this will be the first synthesis of the barriers, supports, and interventions for improving vaccination and HPV vaccination in Indigenous peoples. Synthesis-level knowledge in this area is expected to support Indigenous peoples, researchers, clinicians, policy makers, and communities in their efforts to ameliorate well-documented and disproportionate burdens of disease that persist in Indigenous populations worldwide.

## Methods/design

### Aims

Because we anticipate only a small number of relevant studies on HPV vaccination to emerge from the search, we will employ a broad search strategy to capture literature related to both HPV vaccination and vaccination in general among Indigenous peoples in Canada and globally. This systematic review will locate, gather, and critically appraise the global literature in Indigenous peoples [77], relating to (a) documented barriers and supports to vaccination and specifically HPV vaccination (V/HPV-V) and (b) interventions to increase acceptability and uptake or reduce hesitancy of V/HPV-V.

The design of this mixed methods [82–84] systematic review protocol and search strategy was guided by the Cochrane Handbook for Systematic Reviews [85], the Preferred Reporting Items for Systematic Review and Meta-Analysis (PRISMA) [86] and protocols (PRISMA-P) [87], and ENTREQ [88] reporting guidelines. Because our study will generate both quantitative and qualitative data, we propose segregated analyses on each data set [89]; qualitative data will be managed using NVivo10 [90] and quantitative data will be captured and managed in MS Excel and analyzed using STATA v13.1 [91]. This study protocol was registered with the International Prospective Register of Systematic Reviews on March 1, 2017 (PROSPERO CRD42017048844). Any protocol amendments will be discussed as the review proceeds and documented using a protocol addendum in the final report [92, 93] (see Additional file 1).

### Research questions

Primary questions of interest are:

1. What are the documented barriers and supports to V/HPV-V, in Canadian Indigenous and global Indigenous peoples?
2. What are the documented interventions to increase acceptability and uptake or reduce hesitancy of V/HPV-V, in Canadian Indigenous and global Indigenous peoples?

Secondary questions of interest are:

3. What are the documented barriers and supports to V/HPV-V, in youth (< 18 years) within Canadian Indigenous and global Indigenous peoples?
4. What are the documented interventions to increase acceptability and uptake or reduce hesitancy of V/HPV-V, in youth (< 18 years) within Canadian Indigenous and global Indigenous peoples?

### Eligibility criteria

We will include studies that involve global Indigenous peoples, including First Nations, Inuit, and Métis (Canada), American Indians and Alaskan Natives (USA), and Indigenous peoples in other countries including, but not limited to, China, South Asia, former Soviet Union, Southeast Asia, South America, Africa, Central America/Mexico, Arabia, Japan and the Pacific Islands, Australia, New Zealand, Greenland, and Scandinavia [69, 94, 95]. Study participants may include vaccine recipients, youth, parents, grandparents, guardians, and Elders and/or knowledge holders, health care providers, policy-makers, decision-makers, and community leaders, without restricting by sex or age. Draft inclusion-exclusion criteria were developed (Table 1) and refined using key articles identified from preliminary search findings. Eligible studies will (a) include global Indigenous peoples *and* (b) discuss barriers or supports *and/or* interventions to improve uptake/acceptability or to reduce vaccine hesitancy for V/HPV-V. Studies will be excluded if they do not (a) include global Indigenous peoples or include subpopulation(s) of one or more global Indigenous peoples comprising more than 50% of study participants, or for which separate analyses for global Indigenous peoples or subpopulation(s) are not presented; (b) discuss barriers, supports or interventions for enhancing acceptability or uptake or reducing hesitancy for V/HPV-V; (c) report extractable data; or (d) report original research.

**Table 1:** Study inclusion and exclusion criteria

Inclusion criteria	Exclusion criteria
Global Indigenous peoples (including First Nations, Inuit, and Métis (Canada), American Indians and Alaskan Natives (USA), and Indigenous peoples in other countries including, but not limited to, China, South Asia, former Soviet Union, Southeast Asia, South America, Africa, Central America/Mexico, Arabia, Japan and the Pacific Islands, Australia, New Zealand, Greenland and Scandinavia) [69, 94] and participants may include vaccine recipients, youth, parents, grandparents, guardians, Elders and/or knowledge holders, health care providers, policy−/decision-makers, and community leaders, without restriction on gender or age, AND	Studies that do not include global Indigenous peoples or in which one or more global Indigenous peoples comprise less than 50% of the study participants OR Studies that do not discuss barriers or supports to vaccination or HPV vaccination AND/OR Studies that do not discuss vaccination interventions, or HPV vaccination interventions or enhancing the acceptability or uptake of vaccination or HPV vaccination, or reducing vaccine or HPV vaccine hesitancy OR Studies lacking extractable data (i.e., policy papers or papers with no data) OR
Studies discuss barriers or supports to vaccination or HPV vaccination AND/OR	Non-original research
Studies discuss vaccination interventions to increase acceptability or uptake of vaccination or HPV vaccination, or to reduce vaccine or HPV vaccine hesitancy	

### Outcome measures

Primary outcomes are documented barriers or supports *or* interventions that increase or enhance acceptability/uptake *or* interventions to reduce hesitancy for V/HPV-V. Secondary outcomes will investigate these same factors, specific to youth (< 18 years) in Canadian and global Indigenous peoples. For this review, we define barriers as *single or multilevel factors that are negatively associated with, or hinder the acceptability or uptake of V/HPV-V or increase hesitancy.* Supports include *single or multilevel factors that are positively associated with, or enhance the acceptability and uptake of V/HPV-V or reduce hesitancy*. Vaccination acceptability is defined as *individuals’ willingness to accept vaccination (typically measured by assessing attitudes, beliefs, and/or intention to be vaccinated)* and vaccine uptake is defined as *the behavior of getting vaccinated* [40]. Vaccine hesitancy refers to a *delay in acceptance or refusal of vaccines despite the availability of such services* [41]. In order to optimize the capture of literature, vaccination intervention is defined as *a singular or multilevel, simple and/or complex strategy(ies) used in the design or execution of vaccination or used to increase or enhance vaccine acceptability, uptake or to reduce vaccine hesitancy.* Vaccination is defined as *the act of introducing a vaccine into the body to produce immunity to a specific disease* [96]. Study outcomes will be segregated into three main variable groups: study characteristics, barriers and supports, and vaccination interventions (acceptability, uptake, hesitancy).

### Study type

All study designs meeting inclusion criteria will be considered, without restricting by language, location, or data type (i.e., qualitative, quantitative, and mixed methods studies are eligible).

### Data sources and search strategy

A comprehensive search of the literature will use an a priori search strategy (draft MEDLINE search strategy in Table 1) comprised of key words and controlled vocabulary terms (Medical Subject Headings or MeSH terms). The core search strategy was developed in MEDLINE, in partnership with an academic librarian (HG) and submitted to a second academic librarian (YL) for independent peer review using the Peer Review of Electronic Search Strategies (PRESS) checklist [97]. PRESS feedback was integrated and the finalized strategy translated for use with the remaining databases. We will search several electronic databases (MEDLINE, CINAHL, EMBASE, Web of Science, PsycINFO, Global Index Medicus [LILACS and Western Pacific Region Index Medicus], Cochrane Database of Systematic Reviews, Joanna Briggs Institute Database, Scopus, Bibliography of Native North Americans, University of New Mexico Native Health Database, ProQuest Dissertations, Australian Indigenous HealthInfoNet) from first date of inception to September 2017, without restrictions (Table 2).

**Table 2:** Draft search strategy

Databases: Ovid MEDLINE(R) Epub Ahdead of Print, In-Process & Other Non-Indexed Citations, Ovid MEDLINE(R) Daily and Ovid MEDLINE(R) 1946 to present (Feb 22, 2017)
The core search around the concepts of HPV and vaccination is as follows:
1. exp. Papillomavirus Infections/
2. exp. Vaccination/
3. (hpv or human papilloma* or vaccin* or immuniz*or immunis*).kw,tw.
4. 1 or 2 or 3
To identify studies involving Canadian indigenous populations, we applied a filter developed by health librarians at the University of Alberta [109] to identify Canadian indigenous populations to the above search.
We also ran this search against subject headings and keywords to identify studies related to selected global indigenous populations, as follows:
Databases: Ovid MEDLINE(R) Epub Ahead of Print, In-Process & Other Non-Indexed Citations, Ovid MEDLINE(R) Daily and Ovid MEDLINE(R) 1946 to present (Feb 22, 2017)
1. exp. Papillomavirus Infections/
2. exp. Vaccination/
3. (hpv or human papilloma* or vaccin* or immuniz* or immunis*).kf,tw.
4. 1 or 2 or 3
5. exp. american native continental ancestry group/ or exp. oceanic ancestry group/
6. exp. Health Services, Indigenous/
7. exp. United States Indian Health Service/
8. (aborigin* or Alaska* Native* or American Indian* or Amerindian* or Eskimo* or community-based participatory or indigenous or Inuit* or Maori or Métis or Native American* or Native Hawaiian* or native people* or tribe* or tribal or Pacific Islander* or Torres Strait Islander*).kf,tw.
9. 5 or 6 or 7 or 8
10. 4 and 9

*denotes *wildcard* symbol in the search syntax. The wildcard broadens the search strategy by capturing the denoted word stem and all other derivatives beginning with the same letters

### Gray literature search strategy

A search will be employed to identify original research that is not reported in peer-reviewed journal articles. The search and Level 1 screening process will be conducted concurrently, with two researchers searching a pre-defined group of government, agency, and Canadian and global Indigenous websites to locate relevant gray literature (Table 3). Combinations of the following combinations will be used: (1) Indigenous or Aboriginal or Indian, and (2) Vaccine or Vaccination or Immunization or Immunize or Immunization or Immunize or HPV or Human papillomavirus. Terms will be combined using “and” or “&”. Websites and resources that pertain to (1) Indigenous populations and (2) discuss barriers/supports or interventions to improve vaccination or HPV vaccination will be retained for screening. The title, URL, and data accessed for all websites/resources identified through this search will be entered into an Excel spreadsheet. Questionable resources will be retained for screening. All websites/resources identified in the search will be screened to determine if they meet the inclusion criteria (Table 1). Bibliographic searches will be conducted for all included studies and additional papers identified. We will also contact known provincial, national, and international experts working in this area (e.g., H. O’Donnell [Australia], S. Moore [Australia], R. Bednarczyk [United States]) for advice on relevant published and unpublished works.

**Table 3:** Gray literature – website search plan

Gray literature resource name	URL
Health Sciences Online (HSO)	www.hso.info
OAISter	http://oaister.worldcat.org/
International Network in Indigenous Health Knowledge and Development (INIHKD)	http://www.inihkd.org
Canadian Electronic Library’s Public Documents Collection (previously the Canadian Health Research Collection)	http://www.canadianelectroniclibrary.ca/Default.aspx
Center for World Indigenous Studies	http://cwis.org/
Circumpolar Health Database	http://www.aina.ucalgary.ca/chbd/
Health Info Net	http://www.healthinfonet.ecu.edu.au/key-resources/bibliography
Metis Health Database	https://www.ccnsa-nccah.ca/en/
Native Indigenous Studies Portal	http://iportal.usask.ca/
Public Health Agency of Canada (PHAC)	http://www.phac-aspc.gc.ca/publicat/cdic-mcbc/index-eng.php?utm_source=subscription_list&utm_medium=email_eng&utm_content=index&utm_campaign=CDIC_32_2
First Nations Development Institute	www.firstnations.org
Active Circle	www.activecircle.ca/en
Aboriginal Affairs and Northern Development (Government of Canada)	https://www.aadnc-aandc.gc.ca/eng/1100100010002/1100100010021
Centre for Indigenous Peoples’ Nutrition and Environment (CINE)	https://www.mcgill.ca/cine/centre-indigenous-peoples-nutrition-and-environment-cine
First Nations Environmental Health Innovation Network	http://www.fnehin.ca
Assembly of First Nations	http://www.afn.ca/Assembly_of_First_Nations.htm
First Nations and Inuit Health Branch (Government of Canada – Health Canada)	http://www.hc-sc.gc.ca/fniah-spnia/index-eng.php
National Collaborating Centre for Aboriginal Health (NCCAH) (Government of Canada)	http://www.nccah-ccnsa.ca/en/
National Aboriginal Health Organization (NAHO) (Agency was closed June 29, 2012; archival data will be searched using the Canadian Health Research Collection – archives available on the NAHO website to December 22, 2017)	http://www.naho.ca
Network Environment for Aboriginal Health Research (NEAHR)	http://cahr.uvic.ca/nearbc/elibrary/current-publications/
BC Centres for Disease Control (Chee Mamuk - Aboriginal Health)	http://www.bccdc.ca/our-services/programs/chee-mamuk-aboriginal-health
First Nations in BC Knowledge Network	http://fnbc.info
First Nations Health Authority	http://www.fnha.ca/
Aboriginal Portal	http://aboriginal.ubc.ca/
Indigenous Affairs Website (Government of Alberta)	http://indigenous.alberta.ca
Ministry of Government Relations (First Nations Metis and Northern Affairs) (Government of Saskatchewan)	https://www.saskatchewan.ca/residents/first-nations-citizens/saskatchewan-first-nations-metis-and-northern-affairs-directory
Centre for Aboriginal Health Research (CAHR)	http://umanitoba.ca/centres/cahr/
First Nations, Metis and Inuit Health (University of Manitoba)	http://umanitoba.ca/faculties/health_sciences/medicine/fnmi_health/
Indigenous and Municipal Relations (Government of Manitoba)	https://www.gov.mb.ca/ana/
Ministry of Indigenous Relations and Reconciliation (Government of Ontario)	https://www.ontario.ca/page/ministry-indigenous-relations-and-reconciliation
Centre for Indigenous Studies (University of Toronto)	http://indigenousstudies.utoronto.ca
Secretariat aux affaires autochtones (Gouvernement de Quebec)	http://www.autochtones.gouv.qc.ca/index_en.asp
Aboriginal Affairs (Government of New Brunswick)	http://www2.gnb.ca/content/gnb/en/departments/aboriginal_affairs.html
Office of Aboriginal Affairs (Government of Nova Scotia)	http://novascotia.ca/abor/office/
Aboriginal Affairs Secretariat (Government of Prince Edward Island)	http://www.gov.pe.ca/aboriginalaffairs/
Department of Labrador and Aboriginal Affairs Office (LAAO) (Government of Newfoundland & Labrador)	http://www.laa.gov.nl.ca/laa/
Aboriginal Relations (Government of Yukon)	http://www.eco.gov.yk.ca/aboriginalrelations/
NWT Aboriginal Governments	http://www.daair.gov.nt.ca/en/nwt-aboriginal-governments
Aboriginal Affairs and Intergovermental Relations (Government of the North West Territories)	http://www.daair.gov.nt.ca
Department of Executive and Intergovermental Affairs (Aboriginal Affairs) Government of Nunavut	http://www.gov.nu.ca/eia/information/aboriginal-affairs
Population Research and Outcomes Studies Unit (PROS) (University of Melbourne)	https://health.adelaide.edu.au/pros/
Aboriginal and Torres Strait Islander-Australian Government Policy and Program Connection Website (Government of Australia – and State Site Portal)	http://www.indigenous.gov.au/
Australian Institute of Health and Welfare	http://www.aihw.gov.au/publication-detail/?id=10737419754
Australian Policy Online	http://apo.org.au
The Lowitja Institute (Australia’s National Institute for Aboriginal and Torres Strait Islander Health Research)	http://www.lowitja.org.au
National Centre for Immunization Research and Surveillance (NCIRS) (Government of Australia)	http://www.ncirs.edu.au/
Immunize Australia	http://www.immunise.health.gov.au/
Toi Te Ora Public Health (Government of New Zealand)	https://www.govt.nz/organisations/toi-te-ora-public-health-service/
New Zealand Ministry of Health	https://www.health.govt.nz/
US Centers for Disease Control	http://www.cdc.gov
US Indian Health Service (Federal Health Program for American Indians and Alaska Natives)	https://www.ihs.gov
Immunize Canada	https://immunize.ca/
Ministry of Health-BC	http://www2.gov.bc.ca/gov/content/governments/organizational-structure/ministries-organizations/ministries/health
BC Centre for Disease Control	http://www.bccdc.ca/
Immunize BC	http://www.immunizebc.ca/
Alberta Health	http://www.health.alberta.ca/
Alberta Health Services	http://www.albertahealthservices.ca/
Immunize Alberta	http://immunizealberta.ca/
SK Ministry of Health	https://www.saskatchewan.ca/government/government-structure/ministries/health
Manitoba Ministry of Health, Seniors and Active Living	http://www.gov.mb.ca/health/
Ontario Ministry of Health and long-term care	http://www.health.gov.on.ca/en/
Public Health Ontario	http://www.publichealthontario.ca/en/Pages/default.aspx
Quebec Ministry of Health	http://www.msss.gouv.qc.ca/index.php
Institut national de santé publique du Québec (INSPQ)	https://www.inspq.qc.ca/en
New Brunswick Ministry of Health	http://www2.gnb.ca/content/gnb/en/departments/health.html
Nova Scotia Department of Health and Wellness	https://novascotia.ca/DHW/
Nova Scotia Health Authority	http://nshealth.ca/
PEI Health and Wellness	https://www.princeedwardisland.ca/en/topic/health-and-wellness
Health PEI	http://www.healthpei.ca/
Newfoundland and Labrador Department of Health and Community Services	http://www.health.gov.nl.ca/health/
Canadian Association for Immunization Research and Evaluation (CAIRE)	http://www.caire.ca/about/
Canadian Center for Vaccinology	http://centerforvaccinology.ca/
Vaccine Evaluation Center	http://vaccineevaluationcenter.ca/
Canadian Immunization Research Network (CIRN)	http://cirnetwork.ca/
Yukon Government Department of Health and Social Services	http://www.hss.gov.yk.ca/
NWT Health and Social Services	http://www.hss.gov.nt.ca/
Nunavut Department of Health	http://www.gov.nu.ca/health
Canadian Public Health Association	http://www.cpha.ca/en/default.aspx
Australian Indigenous Health Info Net	http://www.healthinfonet.ecu.edu.au/

### Preliminary findings

A search of key national and international websites that register, fund, or publish knowledge syntheses failed to locate an existing synthesis to address our review questions. We identified several helpful systematic reviews in the areas of vaccine hesitancy [41, 60, 98], acceptability [60, 99], inequity of HPV vaccine uptake [100], barriers and facilitators to HPV vaccination in young women [25, 57, 61, 101, 102], and identification and assessment of interventions to improve HPV vaccine uptake [64, 79, 80, 103]. We identified several tangential emergent syntheses through the PROSPERO International Prospective Register of Systematic Reviews pertaining to parental-reported barriers and facilitators to HPV vaccination [104], hesitancy toward HPV vaccine [105], the effects of mass media interventions on enhancing childhood vaccine uptake [106], health care provider recommendation of HPV vaccine in boys and young men [107, 108], and individual and environmental-level factors influencing HPV vaccination [107]. A relevant review of specific subpopulations that included Canadian or global Indigenous peoples was not located. The core database search was conducted in MEDLINE using the Ovid interface, including in-process and e-pub-ahead-of-print records, without restrictions, and generated a total of 2096 citations after de-duplication. A search filter for identifying literature for Canadian Indigenous peoples was added to the core MEDLINE search and feedback from PRESS review integrated [109].

### Study selection

Search strategy findings will be cataloged using Endnote™ X8.0 and duplicates removed manually. For inclusion, studies must involve one or more global Indigenous peoples *and* discuss barriers or supports to V/HPV-V *and/or* interventions to increase uptake/acceptability or reduce V/HPV-V hesitancy, as per study eligibility criteria. We will exclude studies that do not: a) involve a global Indigenous population (or a subpopulation comprising ≥ 50% of study participants), b) discuss barriers or supports to V/HPV-V; c) discuss V/HPV-V interventions or interventions that enhance the acceptability or uptake of V/HPV-V or reduce V/HPV-V hesitancy, d) report extractable data, or e) report original research.

### Title and abstract screening (Level 1)

Prior to commencing Level 1 (title and abstract) screening [89], we will calibrate screening among reviewers on a random sample (5% of the total number of citations) [83]. All citations will then be screened independently and in duplicate by two investigators [GN/KJM/MSB/NB/SG/CC/RVR] and discrepancies discussed until consensus is reached or, failing consensus, referred to a third reviewer for final resolution. Overall screening concordance will be calculated using the Kappa statistic [110]. Only those studies meeting screening inclusion/exclusion criteria will be retrieved for full text Level 2 [89] review. To pass Level 1 screening, studies must discuss one or more global indigenous peoples *and* barriers or supports to V/HPV-V *or* vaccination interventions to increase acceptability and uptake or reduce hesitancy for V/HPV-V.

### Full-text review and data abstraction (Level 2)

The anticipated outcomes of interest are barriers and supports to V/HPV-V (qualitative and quantitative data, if measured) and interventions to enhance V/HPV-V acceptability *or* uptake or reduce hesitancy (qualitative and quantitative data, if measured), in Canadian and global Indigenous peoples and, secondarily, in Canadian Indigenous youth (< 18 years). Where multiple citations occur, data will be combined to create a full description of the study. Should a lack of clarity about multiple citations arise, information will be sought from authors and studies excluded where discrepancies remain unresolved. We will calibrate full text screening among reviewers using the same methods described for Level 1. The full text of each included citation will be reviewed independently and in duplicate by two investigators [CC/GN/KJM/MSB/NB/SG/SM/RVR/CB] and discrepancies resolved by a third investigator.

A draft data abstraction tool will be generated in MS Excel [111], based on the Data Collection Checklist from the Cochrane EPOC [112]. A subsection of the tool will be developed to assess the included intervention studies, specifically. We will use a set of a priori variables generated from preliminary findings (Table 4), combined with several items adapted from the Community Guide’s Data Abstraction Framework (CGDA) [113] and the Standards for Reporting Implementation Studies Statement (StaRI) [114]. The abstraction tool will be pilot-tested by reviewers on a random sample of included articles and revised until consensus is reached on key variables and definitions.

**Table 4:** Level 2 (full text review) draft data abstraction tool (variables and definitions)

Variable	Definition	
Study characteristics		
Refid	Study unique reference identification number	
Author	Author last name, initials	
Year	Year of publication	
Title	Full title of manuscript	
Language	Original language of publication	
Location	Geographic location (country) of study	
Time	Data collection period (start to end of recruitment)	
Data	Data collection method(s)	
Funding	Reported funding source(s) for study	
Population	Indigenous population, community or ethnicity, as defined by study authors and eligibility criteria as reported by authors	
Target	Target population (health care providers, vaccine recipients, parents, policy makers etc.)	
Report(s)	Source(s) of reported barriers and/or supports	
Number	Number of participants in study	
age_mean, age_sd	Age of included participants [mean (SD), median (IQR), or categorical age, as reported]	
age_med, age-iqr		
age_cat		
Youth	Study participants (or cohort of study participants) were < 18 years (Yes = 1, No = 0, Not Reported NR =99)	
Gender	% female	
Gender_target	Female, male, female and male	
Context	Describe context of implementation (may include a description of the social, economic, policy, healthcare, organizational or other contextual facets that may influence scale implementation)	
Setting	Describe study setting, as defined by study authors (may include characteristics of the locations, personnel, resources for implementation and/or criteria for eligibility as an implementation site that are used in the study	
setting_other	Describe ‘other setting’, as defined by study authors	
Aimobj	Study aims and objectives	
Design	Study design	
	0 = RCT (Randomized controlled trial)	
	1 = NRCT (non-randomized controlled trial)	
	2 = cohort	
	3 = case control	
	4 = controlled before-after	
	5 = interrupted time series	
	6 = qualitative	
	7 = cross-sectional survey	
	8 = scoping, systematic or other synthesis	
	9 = mixed methods	
	10 = other	
design_other	Describe ‘other study design’	
Sampling	Describe sampling strategy	
Inclusion	Study inclusion criteria, as reported by study authors	
Exclusion	Study exclusion criteria, as reported by study authors	
vaccine_delivery	Agency, authority, or group(s) administering vaccine	
vaccine_setting	The environment (clinic, school or other) where the vaccine was delivered	
vaccine_type, vaccine_batch	Type and/or batch of vaccine administered	
vaccine_series	Describe how the vaccine administered (one dose, 3 doses etc.)	
vaccine_status	Reported vaccination status of study participants (Yes = 1, No = 0)	
vaccine_statusdescribe	If yes, describe whether full series, or only partial vaccination etc.	
mod_frame	Authors describe explicitly, a health model, framework or theory utilized to frame their study (Yes = 1, No = 0, Not clear = 99)	
mod_frame_valid	Did the researchers use or test the model/framework or theory? (Yes = 1, No = 0, Not reported = 99)	
mod_framedescribe	Describe model, framework or theory	
Studylim	Reported study limitations	
Onoffres	Location of participants (on reserve, off reserve)	
onoffres_other	Describe location of participants if participants are located off reserve	
Geography	Relative geographic location as reported by study authors	
Ses	Reported socioeconomic status of participants	
education_level	Reported level of education of participants	
knowledge_gen*	Reported participant knowledge about vaccination	
attitudes_gen*	Reported participant attitudes about vaccination	
beliefs_gen*	Reported participant beliefs about vaccination	
behaviors_gen*	Reported participant behaviors regarding vaccination	
knowledge_HPV*	Reported participant knowledge about HPV vaccination	
attitudes_HPV*	Reported participant attitudes about HPV vaccination	
beliefs_HPV*	Reported participant beliefs about HPV vaccination	
behaviors_HPV*	Reported participant behaviors regarding HPV vaccination	
HPV_aware	Awareness of HPV vaccine	
sex_status	Participants reporting sexually active	
pap_status	Participants reporting prior abnormal pap smear test	
STI_status	Participants reporting previous STI	
HPV_status	Participants reporting prior HPV infection	
Barriers and Supports		
barrier*	Describe barriers to vaccine (all types)	subcategories will be determined inductively
barrier_descr	Describe which vaccine type the reported barriers discuss	
barrier_HPV*	Describe barriers to HPV vaccine	subcategories will be determined inductively
support*	Describe supports to vaccine (all types)	subcategories will be determined inductively
support_descr	Describe which vaccine type the reported supports discuss	
support_HPV*	Describe supports to HPV vaccine	subcategories will be determined inductively
recommend_gen*	Describe study recommendations for vaccination (all types)	subcategories will be determined inductively
recommend_descr	Describe which vaccine type the recommendations pertain to	
recommend_HPV*	Describe study recommendations for HPV vaccination	subcategories will be determined inductively
Vaccine Intervention Outcomes		
Outcomeimplem	Describe primary and other outcomes of the implementation strategy, and how they were measured	
Outcomeintervent	Describe primary and other outcomes of the intervention, and how they were measured	
Process	Describe the process evaluation objectives and outcomes related to the mechanism by which the strategy is expected to work	
Intervention*	Describe vaccine intervention	subcategories will be determined inductively
intervention_HPV*	Describe HPV vaccine intervention	subcategories will be determined inductively
implemintervent	Describe the implementation strategy	
HPVimplemintervent	Describe the HPV-specific implementation strategy	
Subgroups	Describe any subgroups or nested studies undertaken in the study	
RR	Risk ratios reported	
RR_pvalue	Reported p-values for RR	
OR	Odds ratios reported	
OR_pvalue	Reported *p*-values for OR	
Econintervent	Methods for resource use, costs, economic outcomes and analysis for the intervention	
Econimplem	Methods for resource use, costs, economic outcomes and analysis for the implementation strategy	
Samplesize	Describe the sample sizes, calculations	
Analysis	Describe analytic methods with rationale for methodological choice	
Subanalysis	Describe subanalyses, representativeness and outcomes of subgroups including those recruited to specific research tasks	
Fidelimplem	Fidelity of the implementation strategy as planned and adaptation to suit context and preferences	
Fidelintervent	Fidelity of delivering the core components of the intervention	
Contextchange	Describe contextual changes that may affect outcomes	
Harms	Describe all important harms or unintended effects for each group	
vaccine_acceptance	Reported factors influencing vaccine acceptance (study participant attitudes and beliefs)	
vaccine_uptake	A rate or quantification of the number or proportion of the study population who received vaccine intervention	
vaccine_refuse	A rate or quantification of the number or proportion of the population who refused vaccine intervention	
vaccine_hesitancy	Describe reported factors relating to under-immunization, delay or questioning of vaccines, selecting only certain vaccines, desire to access a trustworthy healthcare provider [Specific description of hesitancy as a barrier (all types of vaccine)]	
hesitancy_HPV	Describe reported factors relating to under-immunization, delay or questioning of vaccines, selecting only certain vaccines, desire to access a trustworthy healthcare provider [Specific description of hesitancy as a barrier (HPV vaccination)]	
CDGA_IC	CGDA Intervention Category [142] – Informational, Behavioral, Environmental	
CGDA_IL	CDGA Intervention Intensity Level [142] – active engagement to individual, active engagement to population, passive engagement with significant effort, passive engagement with minimal effort	
Interventimplic	Describe policy, practice or research implications for the intervention, including sustainability	
Imlemimplic	Describe policy, practice or research implications for the implementation strategy, specifically related to scalability	
Regulatory	Ethics approval, trial/study registration (and protocol if available), governance of data use, presence of conflicts of interest etc.	

*denotes subcategories will be generated inductively

### Methodological quality

Methodological quality will be assessed using a universal appraisal tool (Quality Assessment Tool for Reviewing Studies with Diverse Designs - QATSDD) developed for the assessment of quality in diverse study designs [115]. The 4-point tool scoring system will be applied to each item, allowing an overall quality assessment for each study using one set of criteria. Team members will independently pilot the quality assessment tool using two to three included studies. The team will review discrepancies and discuss to consensus at a weekly team meeting before proceeding. Two investigators [CC/GN/KJM/MSB/NB/SG/SM/RVR/CB] will assess methodological quality in all included studies, independently and in duplicate. Discrepancies will be noted and discussed until consensus, or failing consensus, referred to a third investigator. Quality scores will be reported as combined reviewer scores across the 16 items and the total score for each study and then summarized and used to fully describe the nature and characteristics of included studies in the analysis. Quality assessment scores will be presented in tabular format for all included studies to highlight the strengths and weaknesses of the evidence base. Quality assessments will be used to 1) describe and characterize the methodological rigor and scientific quality of included studies; and 2) provide context for the interpretation of findings, formulation of conclusions, and/or recommendations arising from the review. Extreme outlier scores will be discussed by the team and depending on the issues identified, may be excluded to avoid the influence of significantly flawed studies on statistical and/or conceptual analyses, as appropriate.

A secondary quality analysis of the included effective intervention studies will be undertaken using the domains of the Consolidated Framework for Implementation Research (CFIR) [116]. The main purpose of this quality analysis is to gather and assess, where available, detail about the presence and nature of the individual-level factors and contextual, intervention or implementation process factors for implementation, using a robust framework.

### Data analysis

The overall study flow will be reported using a PRISMA diagram [86], providing a summary of citation numbers, sources, exclusions/rationale, and final number of included studies. Data analysis of abstracted variables will consist of two main sections with several subsections [82], including: (1) an inductive, qualitative, thematic synthesis [117] of all documented barriers and supports and (2) a quantitative analysis of measured barriers and supports found to be statistically significant, summarized as mean proportion and standard deviation of participants reporting the barrier or support (or other relevant aggregate measures as appropriate) or ranked by frequency of reported barriers or supports. The thematic analysis will proceed using a step-wise, iterative approach. Team members will review each study independently, and in duplicate. Line-by-line coding of identified barriers and/or supports will be conducted on each study, and we will note similarities and differences in codes and discuss any discrepancies to consensus at weekly team meetings. Subsequent studies will be subjected to the same analysis, contributing text to existing (or generating new) themes, as appropriate. Once the coding process is complete, the team will review each of the barrier and support themes and sub themes, refine their definitions if necessary, and examine the text within them for consistency, code interrelations and to identify any potential conceptual hierarchies. All changes to codes, conceptual re-alignments, discussions and decisions will be documented as part of the study audit trail. We will report a summary of emerging themes, sub themes, definitions and linkages to studies in which themes arise.

We anticipate the ability to provide both a descriptive synthesis and quantitative summary of relevant measures for interventions; however, recent reviews examining barriers and supports in the area of HPV vaccination revealed a level of heterogeneity among studies precluding meta-analysis [25]. All included studies describing interventions will be assessed using the Community Guide’s Data Abstraction Framework [118] with interventions categorized and frequencies reported by type and intensity.

We plan to report findings pertaining to Canadian Indigenous and global Indigenous peoples separately, contingent on the volume of eligible literature identified in each group. We will report results for HPV vaccines separately from results arising from studies of other vaccines, given the characteristics and contextual factors associated with HPV compared with other vaccines and target populations. A draft analysis plan and anticipated products of the synthesis are outlined in Table 5.

**Table 5:** Draft analysis plan, analytic method, questions to guide analysis and anticipated review outputs

Review aims	Method	Guiding questions	Anticipated review outputs
Primary Aims			
1: To identify and assess the documented barriers and supports to HPV and other vaccinations in Canadian and global Indigenous peoples	Thematic synthesis	• What are the documented barriers and supports described in eligible studies? • What is the quality of the eligible literature? (QATSDD tool) • What, if any, themes arise in the literature describing barriers and supports to vaccine or HPV vaccine in Canadian and global Indigenous peoples?	• To describe articles that report barriers and/or supports to HPV and other vaccines • To establish the quality of included articles (item and total study scores) • To analyze and (where possible) group barriers and facilitators meaningfully across studies • To analyze any themes among barriers and supports to vaccine or HPV vaccine that are specific to Canadian Indigenous populations • To statistically analyze measured barriers and supports across studies (should they be deemed appropriate for meta-analysis)
	Quantitative Analysis	• What, if any, barriers and supports to vaccine or HPV vaccine in Canadian and global Indigenous peoples are statistically significant?	
2: To identify and assess the documented effective interventions to increase acceptability, uptake or reduce hesitancy of vaccination, and HPV vaccination, in Canadian and global Indigenous peoples	Descriptive statistics, narrative summary	• What interventions are documented to increase vaccine or HPV vaccine acceptability and uptake and reduce hesitancy in Canadian and global Indigenous populations? • What is the quality of the eligible literature? (QATSDD tool) • What, is the nature of vaccine or HPV acceptability, hesitancy and uptake in Canadian and global Indigenous peoples?	• To describe articles that report effective strategies for increasing vaccine or HPV vaccine acceptability, uptake or reducing hesitancy in Canadian and global Indigenous peoples • To establish the quality of included articles (item and total study scores) • To describe quantitatively and qualitatively (thematic synthesis) the nature and characteristics of interventions to increase vaccine or HPV vaccine acceptability and uptake or reducing hesitancy in Canadian and global Indigenous peoples • To synthesize evidence of knowledge, beliefs, and attitudes of Canadian and global Indigenous peoples arising in the eligible literature
	Descriptive Analysis	• What are the features (e.g. intervention category, definition, intensity level) and CFIR domain/subdomain assessment of each reported intervention?	• To describe and systematically assess effective interventions and report findings using the CFIR framework domains/subdomains.
Secondary Aims			
3: To identify and assess documented barriers and supports to vaccination, and HPV vaccination, in youth (< 18 years) within Canadian and global Indigenous peoples.	Thematic synthesis	• What are the documented barriers and supports described in eligible studies for vaccine and HPV vaccine in youth (< 18 years) within Canadian and global Indigenous peoples? • What is the quality of the eligible literature? (QATSDD tool) • What, if any, themes arise in the barriers and supports to vaccine or HPV vaccine in youth (< 18 years) in Canadian and global Indigenous peoples?	• To describe articles that report barriers and/or supports to HPV and other vaccines in Canadian and global Indigenous youth (< 18 years) • To establish the quality of included articles • To analyze (and where possible) group barriers and facilitators meaningfully across studies • To analyze any themes among barriers and supports to vaccine or HPV vaccine that are specific to youth (< 18 years) in Canadian Indigenous populations • Cross-study qualitative synthesis of themes describing barriers and supports
	Quantitative Analysis	• What, if any, measured barriers and supports to vaccine or HPV vaccine in youth (< 18 years) in Canadian and global Indigenous peoples are statistically significant?	• To statistically analyze measured barriers and supports across studies (descriptive statistics; based on recent systematic reviews it is unlikely that a meta-analysis will be possible)
4: To identify and assess documented interventions to increase acceptability and uptake and reduce hesitancy of vaccination and HPV vaccination in youth (< 18 years) within Canadian and global Indigenous peoples.	Descriptive statistics, thematic summary	• What interventions are documented to increase vaccine or HPV vaccine acceptability, uptake and reduce hesitancy in Canadian and global Indigenous youth (< 18 years)? • What is the quality of the eligible literature? • What, if any, themes arise in the descriptions of interventions for vaccine or HPV acceptability/hesitancy and uptake in Canadian and global Indigenous youth (< 18 years)?	• To describe articles that report effective strategies for increasing vaccine or HPV vaccine acceptability, uptake or reducing hesitancy in Canadian and global Indigenous peoples • To establish the quality of included articles • To describe quantitatively (descriptive statistics) and qualitatively (thematic synthesis) the nature and characteristics of interventions to increase vaccine or HPV vaccine acceptability, uptake or reduce hesitancy in Canadian and global Indigenous youth (< 18 years) (based on recent systematic reviews it is unlikely that a meta-analysis will be possible) • To synthesize Canadian and global Indigenous youth (< 18 years) knowledge, beliefs and attitudes arising in the eligible literature • Cross-study qualitative synthesis of themes describing acceptability, uptake and reduced hesitancy interventions
	Descriptive Analysis	• What are the features (e.g., intervention category, definition, intensity level) and CFIR domain/subdomain assessment of each reported intervention to increase acceptability and uptake, and reduce hesitancy of vaccination and HPV vaccination in youth (< 18 years) within Canadian and global Indigenous peoples?	• To describe and systematically assess interventions to increase acceptability and uptake, and reduce hesitancy of vaccination and HPV vaccination in youth (< 18 years) within Canadian and global Indigenous peoples, and report findings using the domains/subdomains of the CFIR framework.

## Discussion

To date, we have not located a systematic review describing the barriers and supports to HPV vaccination or one that gathered and assessed effective interventions to increase acceptability and uptake of HPV vaccines in Indigenous peoples. Previous systematic reviews have been limited to vaccine efficacy in Indigenous peoples [119–121]. In addition to identifying studies that describe the barriers and supports to vaccination and interventions to increase vaccination acceptability and uptake in Indigenous peoples, the proposed systematic review will also aim to identify studies that specifically describe the barriers and supports to HPV vaccination and interventions that have been shown to increase acceptability and uptake of the HPV vaccine. While there is some evidence to suggest that tailoring vaccine strategies to the needs and preferences of adolescents using technology interventions may be promising, it is unclear how these findings relate to Canadian Indigenous youth. We have chosen to focus on HPV vaccination because, despite wide-spread availability, uptake of HPV vaccination between Indigenous and non-Indigenous youth remains unequal [19]. This finding is similar to that found in non-Indigenous populations in the USA where only 65% of girls and 56% of boys initiate a HPV vaccination series and fewer (43% and 32%, respectively) complete the series [64]. The approach we propose will allow us to synthesize existing evidence of HPV vaccine-specific barriers, supports, and effective interventions that may provide insights into vaccination practices that better meet the needs of Indigenous peoples.

While the importance of multiple [79, 80, 122] and/or tailored [123, 124] (versus singular) interventions in some target groups remains a matter of debate in the implementation literature [125], multiple strategies focused on increasing HPV vaccination rates in non-Indigenous peoples have been shown to be effective [80, 126–132]. Furthermore, interventions tailored to address specific barriers to HPV vaccination or to address contextual differences may be required [133, 134]. It is unlikely, given the apparent state of the literature, that detailed information is available about contextual influences on HPV vaccination barriers and supports and HPV vaccination acceptability/hesitancy and uptake.

We have chosen to include global Indigenous peoples [133, 134] and anticipate that this review may suggest that Indigenous peoples, regardless of country, may experience similar barriers and supports to vaccination acceptability/hesitancy and uptake. With this in mind, we chose a broad definition of Indigenous peoples to help generate results that are relevant to many stakeholders. Given the dearth of literature in this area, we will consider all study designs meeting inclusion criteria, without restriction by language, location, or data type. We anticipate only a small number of relevant studies to emerge from the search and, hence, have used a broad, less restrictive search strategy that aims to capture literature related to V/HPV-V among Indigenous peoples.

The results of this study are expected to inform future research, policies, programs, and community-driven initiatives to enhance acceptability and uptake of vaccination among Indigenous peoples. Specifically, this review will identify and describe the documented barriers, supports, and interventions for V/HPV-V. These will provide a foundation to inform the development of new vaccination strategies among Indigenous peoples. We anticipate the review will identify the nature of evidence gaps and highlight areas requiring further study.

### Dissemination and knowledge translation plan

To our knowledge, this study will contribute the first systematic review of the global literature in this area. We will take an integrated knowledge translation [135, 136] approach to guide our synthesis in close partnership with First Nation knowledge holders and others who will help interpret, structure, and disseminate findings to relevant research, clinical, Indigenous, policy, and decision maker groups. We anticipate there may be important regional, group, and contextual differences present in the data that will require tailored dissemination (EHVINA Study Research Team: Alberta First Nations Health Care Professional Stakeholder Meeting (2015), unpublished; EHVINA Study Research Team: EHVINA study – Alberta First Nations Elders Gathering (2015), unpublished; EHVINA Study Research Team: Canadian Institutes for Health Research - Institute for Cancer Research Stakeholder Engagement Brainstorming Session (2017), unpublished; EHVINA Study Research Team: Exploring International Collaborative Connections (2016), unpublished) and plan to co-present findings with First Nation partners. We anticipate strong receptivity for study findings and will utilize a translation process that is informed by the Knowledge-to-Action framework [137, 138] but is deliberately inductive to ensure knowledge systems, contexts, and the unique needs of our knowledge users are considered [77]. This process will extend from the analysis and interpretation of review findings through the conceptualization, mapping, and execution of vaccination strategies [139–141]. In closing, the intent of this synthesis is to assess the global literature pertaining to barriers and supports to V/HPV-V and identify effective interventions to enhance acceptability and uptake of V/HPV-V, in Canadian and global Indigenous peoples. We anticipate that this synthesis will provide numerous stakeholder groups with a better understanding of the current state of the literature in this area and help inform the development of interventions that encourage V/HPV-V acceptability and uptake and reduce hesitancy in Canadian and global Indigenous peoples.

## Additional file

Additional file 1: PRISMA-P Checklist. (DOCX 39 kb)

## Abbreviations

CDGA: Community guide’s data abstraction framework (CGDA) (https://www.thecommunityguide.org/about/our-methodology)
CFIR: Consolidated framework for implementation research
ENTREQ: Enhancing transparency in reporting the synthesis of qualitative research: ENTREQ
EPOC: Cochrane effective practice and organization of care (epoc.cochrane.org/)
FN: First nations
HPV: Human papilloma virus
KT: Knowledge translation
NVivo: qualitative data analysis software from QSR international (www.qsrinternational.com/nvivo/what-is-nvivo)
PRESS: Peer review of electronic search strategies: 2015 guideline statement
PROSPERO: International prospective register of systematic reviews (https://www.crd.york.ac.uk/prospero/)
QATSDD: Quality assessment tool for reviewing studies with diverse designs
STaRI: Standards for reporting implementation studies statement
STATA: Data analysis and statistical software program (https://www.stata.com/)
URL: Universal resource locator
USA: United States of America
V/HPV-V: Vaccination and specifically HPV vaccination
